# Cytotoxicity Evaluation of High-Temperature Annealed Nanohydroxyapatite in Contact with Fibroblast Cells

**DOI:** 10.3390/ma10060590

**Published:** 2017-05-27

**Authors:** Maria Szymonowicz, Mariusz Korczynski, Maciej Dobrzynski, Katarzyna Zawisza, Marcin Mikulewicz, Ewa Karuga-Kuzniewska, Boguslawa Zywicka, Zbigniew Rybak, Rafal J. Wiglusz

**Affiliations:** 1 Department of Experimental Surgery and Biomaterial Research, Wroclaw Medical University, Poniatowskiego 2, 50-326 Wroclaw, Poland; maria.szymonowicz@umed.wroc.pl (M.S.); mariusz.korczynski@upwr.edu.pl (M.K.); zbigniew.rybak@umed.wroc.pl (Z.R.); 2 Department of Environment Hygiene and Animal Welfare, Wroclaw University of Environmental and Life Sciences, Chelmonskiego 38c, 51-630 Wroclaw, Poland; boguslawa.zywicka@umed.wroc.pl; 3 Department of Conservative Dentistry and Pedodontics, Wroclaw Medical University, Krakowska 26, 50-425 Wroclaw, Poland; maciej.dobrzynski@umed.wroc.pl; 4 Institute of Low Temperature and Structure Research, Polish Academy of Sciences, Okolna 2, 50-422 Wroclaw, Poland; k.zawisza@int.pan.wroc.pl; 5 Department of Dentofacial Orthopeadics and Orthodontics, Division of Facial Abnormalities, Wroclaw Medical University, Krakowska 26, 50-425 Wroclaw, Poland; mikulewicz.marcin@gmail.com; 6 Department of Epizootiology and Clinic of Bird and Exotic Animals, Wrocław University of Environmental and Life Sciences, Pl. Grunwaldzki 45, 50-366 Wrocław, Poland; ewa.karuga-kuzniewska@upwr.edu.pl

**Keywords:** nanohydroxyapatite, biodegradable, fibroblasts cells, cytotoxicity test

## Abstract

Biomaterials are substances manufactured for medical purposes in direct contact with the tissues of organisms. Prior to their use, they are tested to determine their usefulness and safety of application. Hydroxyapatites are used in medicine as a bony complement because of their similarity to the natural apatite therein. Thanks to their bioactivity, biocompatibility, stability and non-toxicity hydroxyapatite are the most commonly used materials in osteoimplantology. The use of materials at the nanoscale in medicine or biology may carry the risk of undesirable effects. The aim of the study was to evaluate the cytotoxic effect of high-temperature annealed nanohydroxyapatites on the L929 murine fibroblasts. Nanohydroxyapatites in powder form were obtained by the wet chemistry method: in the temperature range of 800–1000 °C and used for the study. Based on performed studies evaluating the morphology and fibroblast viability, it was found that nanohydroxyapatites show no cytotoxic effects on the L929 cell line.

## 1. Introduction

The demand for new biomaterials continues to grow due to the increasing average life expectancy, traumatism, lowering the age of the users, and the expectation of continuous improvements in the quality of life [1,2,3,4]. The range of biomaterial use is wide. Depending on the intended use of the material, it must have specific properties. Biomaterial used for bone substitution should be a safe material with a composition as close as possible to the natural bone tissue, which it is meant to replace. In analyzing the chemical composition of the bone system, it was found that the bones are composed of 30% organic and 70% inorganic substance, such as apatite (approx. 65% bone mass) [5,6,7]. In the organism, there is a hydroxyapatite with a molar ratio of Ca/P equalling at least 1.67, crystallizing in a hexagonal system [6]. To create the ideal material for use in bone surgery and dentistry, hydroxyapatite was introduced to synthesize obtaining stoichiometric bioceramics. Its advantages include a high degree of biocompatibility, osteoinductivity and osteointergrity [5,8,9,10]. Hydroxyapatite is obtained in two forms—compact and porous. The first form is characterised by low biodegradability, but with a high coefficient of brittle fracture. Therefore, its use is limited to implants not transmitting high stress. The second form is resorbable hydroxyapatite, which is gradually replaced by regenerating bone tissue [9,11]. Hydroxyapatite is used as an individual bone substitute biomaterial, as coating on other materials, e.g., metal grafts, or as a component of composite materials. The use of hydroxyapatite as coating results in improved biological properties of the medical device. The addition of hydroxyapatite to composite materials results in obtaining better mechanical and biological properties of the designed product [11,12].

In the Institute of Low Temperature and Structure Research Polish, Academy of Sciences in Wroclaw, they have developed a method of obtaining hydroxyapatite nano-scale that has affected the change of the properties of the obtained material in relation to the hydroxyapatite obtained so far [7]. A number of different strategies for the synthesis of nanoparticles based on such techniques as microemulsion, coprecipitation, thermal decomposition and others have developed. However, the best results and control of the grain/particle sizes, the degree of crystallization and phase purity can be obtained using methods based on wet chemical synthesis [13]. Using the above methods, the group R.J. Wiglusz obtained apatite nanomaterials Ca_10_(PO_4_)_6_OH_2_ for biomedical applications with a high degree of crystallization and phase purity but also with high homogeneity and a relatively low degree of agglomeration of grains [8]. Another important factor from the viewpoint of bio-applications is the high biocompatibility of the proposed materials additionally based on the synthesis without the use of toxic solvents. Research on admixing nanohydroxyapatite (nHAP) with metal ions are being conducted [7]. Particle size of the nHAP (40–80) nm affects the possibility to use this material for anticancer, antimicrobial therapy and as a drug carrier [6,14,15]. Nanomaterials are a rapidly developing branch of medicine due to the fact that their size, in at least one dimension, is similar to antibodies, membrane receptors, and nucleic acids. This feature allows you to use them as carriers of drugs or imaging diagnostics [16]. The particle size affects the appearance of the risk of combining with the cell membrane, permeation through it into the cell, and interfering with its function, even when the nanoparticles are inert [15,16,17]. There are still no legal regulations concerning the handling of materials at the nanoscale, as well as specific procedures for evaluating the toxicity of nanoparticles [16]. For this reason, it is extremely important to study nanomaterials in terms of biocompatibility and to determine the suitability and safety of the use of biomaterials in medicine. The study of in vitro cytotoxicity is one of the methods that allows for determining the impact of the test material on cell lines. It allows biological pre-screening evaluation of biomaterials for possible toxic effects of the substances washed out from the material. High sensitivity of these tests is the result of the isolation of cell cultures and, therefore, the lack of defence mechanisms that accompany cells in the organism.

The aim of the study was to determine the cytotoxic effect of nanohydroxyapatites through the determination of morphological changes and cell viability of mouse fibroblast cells L-929 in vitro tests.

## 2. Results and Discussion

### 2.1. X-ray Powder Diffraction

**Figure 1:**
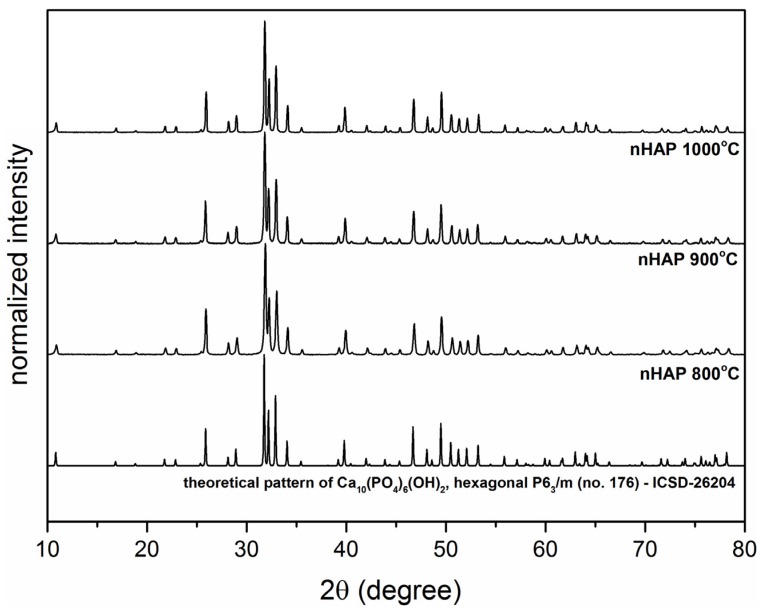
X-ray powder diffraction patterns of nHAP powders annealed at a temperature range of 800 to 1000 °C and a theoretical pattern of hexagonal Ca_10_(PO_4_)_6_(OH).

The formation of the crystalline hydroxyapatite nanopowders was followed by the XRD (X-ray Diffraction) measurements (see Figure 1). All of the samples prepared utilizing co-precipitation techniques shown detectable crystallinity at all ranges of proposed sintering temperatures (800–1000 °C). Comparison of the resulting diffraction patterns with the reference standard of the Ca_10_(PO_4_)_6_(OH)_2_ (ICSD-26204) [18] confirms the presence of single phase, hexagonal structure (P6_3_/m (No. 176)) of the final products. An increasing of the average grain size is caused by the calcination temperature, as a result of the well-known Ostwald ripening process. Depending on the sintering temperature, the crystallize size was estimated to be 45 nm for the sample sintered at 800 °C, 63 nm for the sample obtained at 900 °C and 82 nm for the sample annealed at 1000 °C, indicating progressive grain growth.

### 2.2. TEM Microscopy

**Figure 2:**
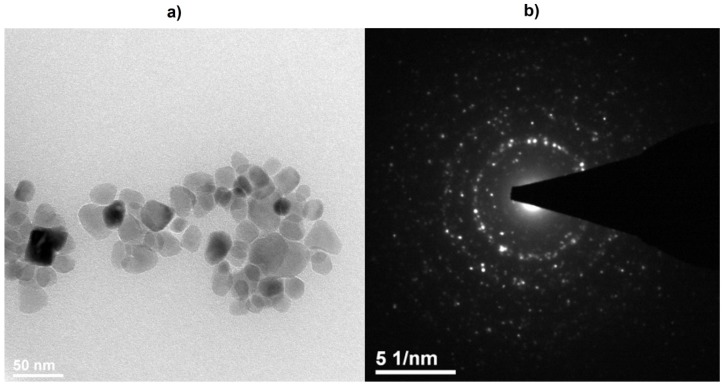
Selected TEM (**a**) and SAED (Selected Area Diffraction) (**b**) images of the nHAP nanoparticles obtained at 800 °C.

The final confirmation of particle size of the nHAP powders was done utilizing HRTEM microscopy (High Resolution Transmission Electron Microscopy, Philips CM-20 Super Twin microscope, Eindhoven, The Netherlands). In accordance with the TEM study (see Figure 2), the particles of Ca_10_(PO_4_)_6_(OH)_2_ sintered at 800 °C are regular with a mean particle size of 40 nm. Analysis of SAED (Selected Area Diffraction) pattern revealed the appearance of well developed rings with clear reflections at positions corresponding with a reference standard of calcium hydroxyapatite.

### 2.3. ICP-OES Analysis

Element analysis was done using the ICP-OES (Inductively Coupled Plasma—Optical Emission Spectrometers, Agilent 720 apparatus, Santa Clara, CA, USA)) technique (Table 1) in order to confirm the composition and homogenous distribution of the Ca and P. All of the constituting elements were in a proper molar ratio confirming right stoichiometry of the final material. The ratio of the Ca^2+^ cation to the P^5+^ was about 1.67 for all samples, well matching with the theoretical ratio of Ca/P in calcium hydroxyapatite.

### 2.4. Cytotoxicity

**Figure 3:**
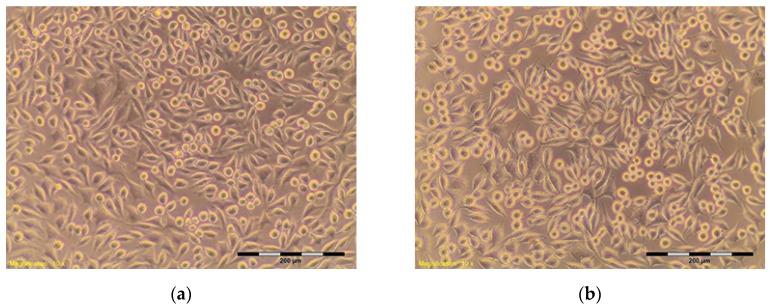
L-929 cells—blind test (**a**,**b**). Contrast reverse-phase microscope CKX41 (Olympus), mag. 10×.

**Figure 4:**
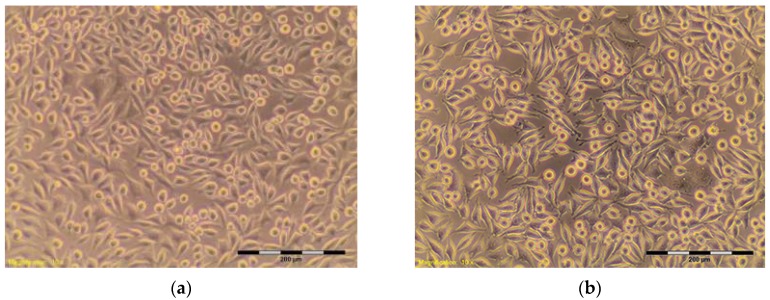
L-929 cells after contact with HDPE—negative control (**a**,**b**). Contrast reverse-phase microscope CKX41 (Olympus), mag. 10×.

**Figure 5:**
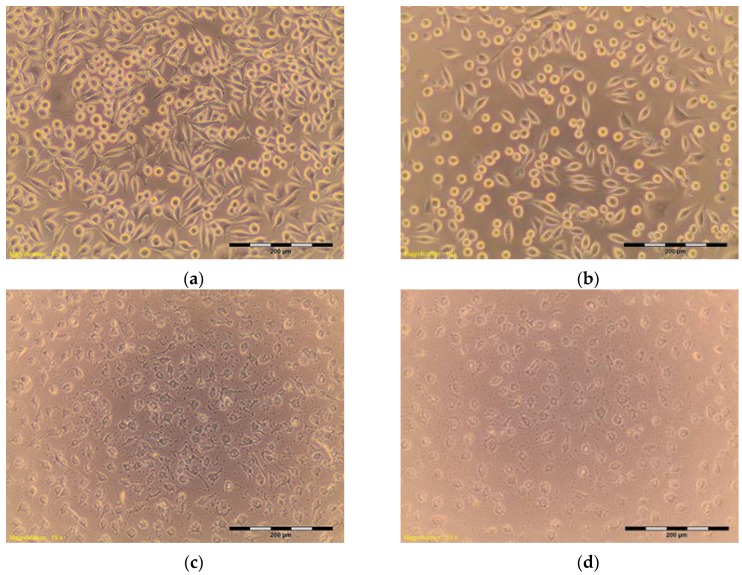
L-929 cells after contact with: (**a**) SLS (Sodium Lauryl Sulfate) 0.05 mg/mL; (**b**) SLS 0.10 mg/mL; (**c**) SLS 0.15; (**d**) SLS 0.2 mg/mL—positive control. Contrast reverse-phase microscope CKX41 (Olympus), mag. 10×.

**Figure 6:**
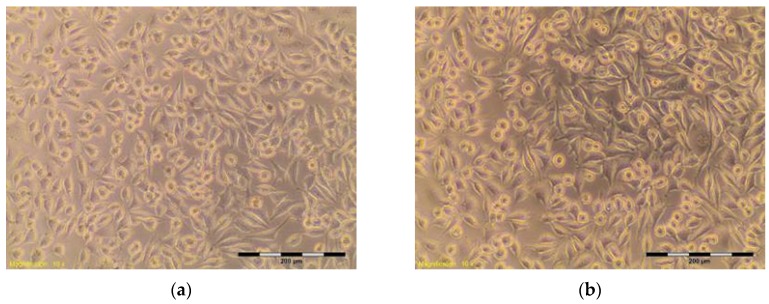

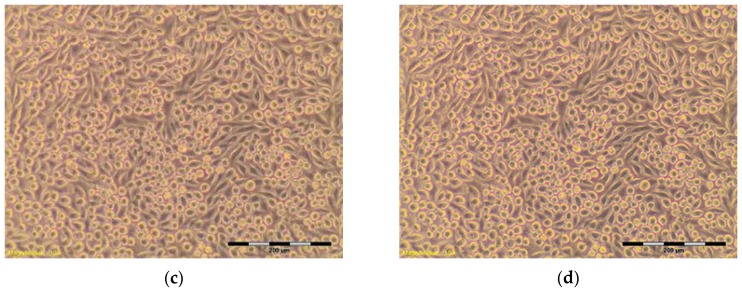
L-929 cells after contact with nHAP-800 suspension: (**a**) 100%; (**b**) 50%; (**c**) 25%; (**d**) 12.5%. Contrast reverse-phase microscope CKX41 (Olympus), mag. 10×.

**Figure 7:**
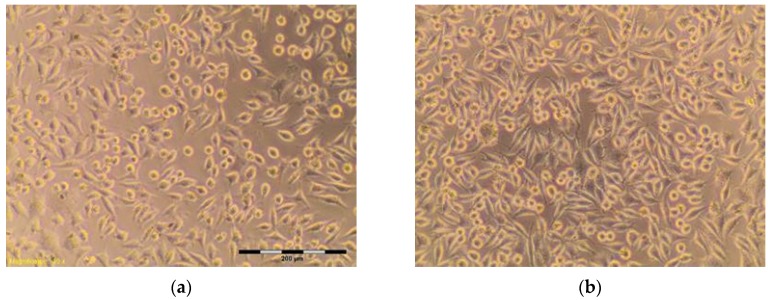

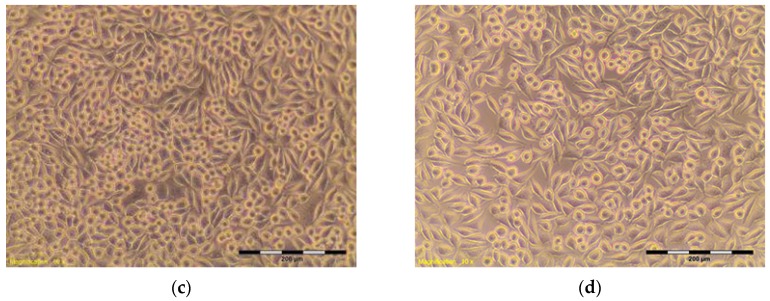
L-929 cells after contact with nHAP-900 suspension: (**a**) 100%; (**b**) 50%; (**c**) 25%; (**d**) 12.5%. Contrast reverse-phase microscope CKX41 (Olympus), mag. 10×.

**Figure 8:**
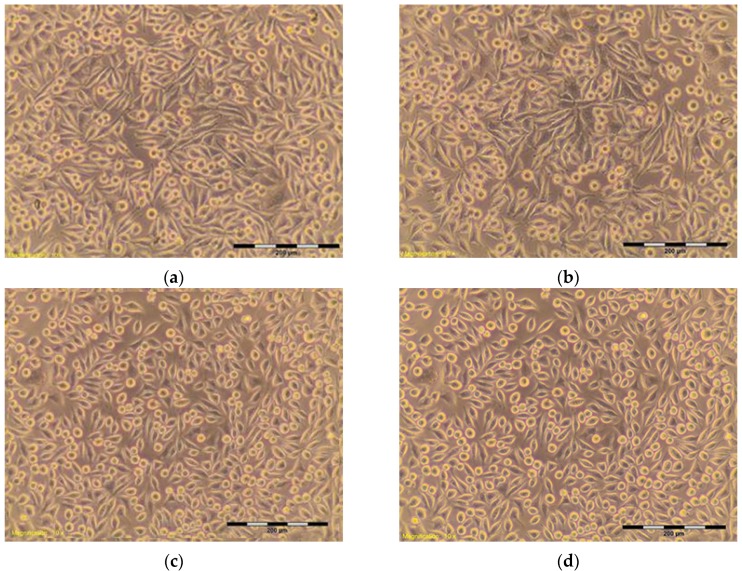
L-929 cells after contact with nHAP-1000 suspension: (**a**) 100%; (**b**) 50%; (**c**) 25%; (**d**) 12.5%. Contrast reverse-phase microscope CKX41 (Olympus), mag. 10×.

The percentage of survival and evaluation of changes in the morphology of mouse fibroblast cells L-929 (Figure 3) after contact with suspensions from the control materials (HDPE, SLS) and test materials from nHAP-800, nHAP-900 and nHAP-1000 are given in Table 2 and Table 3. Microscopic images of the morphology of cell cultures treated with the suspensions from nanohydroxyapatites and the control are shown in Figure 4, Figure 5, Figure 6, Figure 7 and Figure 8. Evaluation of nanohydroxyapatite, regardless of the temperature used in the production process, that is, nHAP-800, nHAP-900 and nHAP-1000, did not reduce L-929 cell survival below 80%. No significant differences were observed in the survival of cells in the suspensions 100% between nHAP-800, nHAP-900 and nHAP-1000. L-929 cell survival after contact with suspension from material nHAP-800 was 87.64%. In the case of materials nHAP-900 and nHAP-1000, the percentage of survival rate was, respectively, 88.87% for nHAP-900 and 88.30% for nHAP-1000. With a reduced concentration of suspensions, increase in cell survival was observed. The highest values were observed for nHAP-1000; cell survival increased on average by 21%.

In a microscopic image of L-929, cells treated with the suspension 100% from nHAP-800, the cytoplasmic granules were observed inside. Approximately 10% of the cells in the culture shrunk and became detached from the substrate (Figure 6); those changes were classified by a criterion for degree 1—weak toxicity. Similar changes in cell morphology were observed in systems with 100% of the applied suspension—from nHAP-900 and nHAP-1000. Cells in the range of 10–15% shrunk and became detached from the substrate, and the appearance of fine granules inside cytoplasmic cells was observed (Figure 7a and Figure 8a). With reduced concentration of suspension from nanohydroxyapatite single intraplasmic granules, culture density comparable to the density of the control culture was observed. There were a lot of cells in the divisions, single cells shrunk, and no cell lysis was observed. Reducing the concentration of the suspension from nanohydroxyapatite to 25%, single intra cytoplasmatic granulations were observed, and culture density was comparable to the densities of the control culture. There were a lot of cells in the divisions, single cells were shrunk, and no cell lysis was observed. Regardless of the material used, there was no significant change in the amount of L-929 cells in comparison to the control.

In surgical practice, for the reconstruction of bone defects synthetic hydroxyapatite in chemical formula Ca_10_(PO_4_)_6_(OH)_2_, which acts as a substitute for bone tissue, is used on an increasing scale. The chemical composition and crystalline structure of synthetic hydroxyapatite are similar to the mineral component of bone. The hydroxyapatite is used in the powder form (or paste) to fill bone defects, in the form of ceramic fittings, or in the form of coatings applied to metal implants (e.g., hip or knee endoprostheses made from titanium alloys). Hydroxyapatite implanted into a living organism is not resorbable by this organism; however, it fills an existing gap in the bone or forms a bioactive layer between the metal implant and the bone. The bioactivity of hydroxyapatite consists of that on its surface, and, as a result of activity of bone-forming cells (osteoblasts), a new natural bone tissue will grow.

Many studies of in vitro cytotoxicity of hydroxyapatite are examined. The hydroxyapatite in the form of granules was tested with neutral red uptake assay NRU (neutral red solution) and the colorimetric MTT assay (3-[4,5-dimethylthiazol-2-yl]-2,5-diphenyl tetrazolium bromide). In the study, it showed no toxicity to human fetal hFOB osteoblasts, and their survival rate was about 70% in relation to the control after 24 h and after 48 h of the study. In the MTT, significant differences in the results were observed depending on the prepared suspension from hydroxyapatite. The suspension from highly porous granules showed no toxicity, and cell viability was 70.6% and 60.3% compared with the control assay after 24 and 48 h of incubation. In the case of suspension from microporous hydroxyapatite, granules showed a decrease in cell viability to 52.4% after 24 h and 37.7% after 48 h of incubation [19]. The authors assumed that the result was due to suspension ion, during which changes to the ions concentrations occurred in the culture medium, which was not without an effect on the metabolism of the cultured cells. Analysis of the results confirmed that the addition of hydroxyapatite to other materials has an impact on cell viability and metabolism [19]. Another form of the hydroxyapatite matrices (scaffolds) was also studied, on which the cells were applied. Scaffolds were made of composite PLGA (poly(lactic-co-glicolic acid) or composite PLGA-HAP. The MTT assay showed that the viability of cells cultured on the scaffold from PLGA was lower than in the case of the PLGA-HAP scaffold [20].

Hydroxyapatite has very good biological properties. For this reason, more and more biological research is conducted on nanohydroxyapatite. In studies carried out by Chen et al., the assay MTT and LDH (lactate dehydrogenase) test to evaluate the activity of lactate dehydrogenase on murine cells MC3T3 E1 preosteoblasts [21]. The test material was nanohydroxyapatite positively, negatively charged and neutral. The tests showed improved survival and proliferation of cells in relation to the control system—polystyrene. Positively charged nanoparticles of hydroxyapatite were characterized with the highest improvement in survival and proliferation of cells [21]. Lack of cytotoxic activity in relation to cells of mouse fibroblasts of line L-929, demonstrated in our own study in vitro, allows for qualifying the evaluated nanohydroxyapatites to further stages of biological evaluation. Depending on the future use of nanohydroxyapatite, it is necessary to conduct, among others, a study of allergenic and irritating reactions, as well as an evaluation of reactions after implantation. Obtaining negative results in tests, in vitro proves that the developed nanomaterials have no toxic effect and can be used in the future as materials for biomedical applications.

## 3. Materials and Methods

Nanohydroxyapatites in powder form, nHAP-800, nHAP-900 and nHAP-1000, obtained by the wet chemistry method at temperatures of 800 °C, 900 °C, and 1000 °C, respectively, were used for the study [7]. Materials were designed and manufactured in the Institute of Low Temperature and Structure Research, Polish Academy of Sciences, Wroclaw, Poland. The cytotoxicity tests were carried out on the reference mouse fibroblast cell line L-929 (Figure 3). The investigation was conducted at the Department of Experimental Surgery and Biomaterials Research, Wroclaw Medical University, Poland [12,13,14].

### 3.1. Apparatus

Evolution of crystal structure was followed using the XRD technique by collecting patterns in 2θ range of 5°–120° with X’Pert PRO X-ray diffractometer (Cu, Kα1: 1.54060 Å) (PANalytical, Almelo, The Netherlands). The morphology and microstructure of nanoparticles were investigated by high-resolution transmission electron microscopy (HRTEM) using a Philips CM-20 Super Twin microscope (Eindhoven, The Netherlands), operated at 200 kV. Samples for measurements were prepared by dispersing of powders in methanol. Afterwards, a droplet of suspension was deposited on a copper microscope grid covered with perforated carbon. ICP-OES elemental analysis was done using an Agilent 720 apparatus (Santa Clara, CA, USA). Calibration curves were measured using ICP standard solution for determination of the Ca^2+^ and P^5+^ ions content. Prior to elemental analysis, the nHAP samples were digested using HNO_3_ with spectral purity.

### 3.2. Synthesis of Ca_10_(PO_4_)_6_(OH)_2_ Nanoparticles (nHAP)

The main substrates used for fabrication of the Ca_10_(PO_4_)_6_(OH)_2_ nanoparticles (nHAP) using a co-precipitation technique were Ca(NO_3_)_2_·4H_2_O (99.98% Alfa Aesar, Karlsruhe, Germany) and (NH_4_)_2_HPO_4_ (99.99% Sigma Aldrich, Saint Louis, MO, USA) as well as NH_4_OH (99% Avantor Performance Materials Poland S.A., Gliwice, Poland) for pH control.

In this method, stoichiometric amounts of Ca(NO_3_)_2_·4H_2_O and (NH_4_)_2_HPO_4_ were dissolved in MQ-water separately. Subsequently, the prepared solutions were mixed. As a result, there was fast precipitation of the by-product. The solution pH was adjusted to 10 with NH_4_OH under constant and vigorous stirring at 90 °C for 2 h. Finally, the by-product was dried for 24 h at 90 °C and thermally treated at the temperature range of 800–1000 °C for 3 h, resulting in formation of white, fine-grained powders.

### 3.3. Cell Culture

Murine fibroblast cells L-929 (NCTC clone 929: CCL 1, American Type Culture Collection ATCC^®^) were cultured in Eagle’s Minimum Essential Medium (EMEM) with L-glutamine (ATCC^®^) and supplemented with 10% foetal bovine serum (FBS, Lonza^®^). L-929 is a cell line recommended by ISO 10993-5 for cytotoxicity assessment of biomaterials. The cells were cultured in a standard condition (5% CO_2_, (37 ± 1) °C) in a Steri Cycle 381 (Thermo Scientific, Marietta, OH, USA) incubator.

### 3.4. Suspension Preparation

Suspensions of the materials investigated (nHAP-800, nHAP-900 and nHAP-1000) and negative control materials were prepared under sterile conditions in proportion: samples 20 g/mL in culture medium. As a negative control, high density polyethylene (HDPE U.S. Pharmacopeia—Rockville, MD, USA) was used, and, as a positive control sample, a solution of sodium lauryl sulfate (SLS, Sigma-Aldrich^®^, St. Louis, MO, USA) with indicated concentrations (0.1, 0.15, 0.2) mg/mL was used. To evaluate the cytotoxicity, the following concentrations of suspensions were used: 100%, 50%, 25% and 12.5%. Blank (complete cell culture medium without any samples) was also included.

### 3.5. Cytotoxicity Tests

Murine fibroblast cells L-929 (NCTC clone 929) were obtained from ATCC were cultured in medium Minimal Essential Eagle’s Medium (MEM) (Lonza) supplemented with 10% FBS (Fetal Bovine Serum) and L-glutamine with streptomycin and penicillin solution (Sigma-Aldrich^®^). Cells were removed from culture flasks with the use of 0.25% trypsin-EDTA (ethylenediaminetetraacetic acid) solution (Sigma-Aldrich^®^) and seeded in 96-well flat-bottomed plates (Nunc, Nunclon^™^ Surface Roskilde, Denmark) in a concentration of 1 × 10^4^ cells per well (1 × 10^5^ cells per mL). After 24 h of incubation in standard conditions, the cells medium was discarded and replaced with 100 µL of suspension or control. The evaluation of the cytotoxic effect was provided after 24 h incubation at (37 ± 1) °C in a humidified atmosphere containing 5% CO_2_ with suspension and included the determination of morphological changes and viability of the cells. The changes in cell morphology were evaluated with the use of the contrast reverse-phase microscope CKX41 (Olympus, Tokyo, Japan), according to the criteria given in Table 4, According to the 4-grade scale, changes in the cell cultures higher than 2 degrees and a reduction of cell viability greater than 30% are considered to be caused by the cytotoxic effect [12]. The cell viability was determined by MTT assay with concentration of 3-4,5-dimethylthiazol-2-yl]-2,5-diphenyl tetrazolium bromide (MTT) (Sigma-Aldrich^®^) 1 mg/mL in EMEM (ATCC^®^). To provide the MTT assay, the suspensions of samples were discarded and 50 µL of MTT solution was added to each well and plates were incubated for 3 h at (37 ± 1) °C in 5% CO_2_. Then, the MTT solution was discarded and 100 µL of isopropanol, analytical grade (Stanlab^®^ Lublin, Poland) were added in each well. After 30 min, the absorbance values were recorded at 570 nm using an Epoch microplate spectrophotometer (BioTek^®^ Highland Park, MI, USA). Cell viability was calculated according to formula (1):

*V* = (*A*b:*A*s) 100%, (1)

where *V* is cell viability percentage, *A*b is mean absorbance of the test sample, and *A*s is mean absorbance of the blank.

## 4. Conclusions

The high-temperature annealed nanohydroxyapatites (nHAP) were obtained using a co-precipitation technique and heat-treated in the temperature range of 800–1000 °C. The detailed study of the hydroxyapatite structures presented complete crystallization and was confirmed by X-ray diffraction and TEM analysis. All of the samples caused only a slight reduction of cell viability. The cell viability (MTT test) and the evaluation of the morphology of cells that were treated with the extracts of the nHAPs showed the low (grade 1) toxicity of the tested materials. In accordance with the norm ISO 10993-5, changes in the culture above the second degree of toxicity reduction of cell viability by more than 30% and morphological changes in cells (intracytoplasmic granules, not more 50% growth inhibition, 50% of cells are round) are considered a cytotoxic effect. Taken together, investigated materials on a 4-grade scale of cytotoxic effect cause 1 degree and are considered a slight cytotoxic. The examined nanohydroxyapatites have not shown cytotoxic effects on L-929 murine fibroblasts.

## Figures and Tables

**Table 1:** Results of the ICP-OES * analysis of the nHAP nanoparticles.

Sample Mass (g)	Sample nHAP	Ca (mg/mL)	P (mg/mL)	Ca (mol)	P (mol)	Ca/P
0.1	800 °C	382.5	176.5	0.9544	0.5698	1.675
	900 °C	380.6	175.6	0.9496	0.5669	1.675
	1000 °C	378.8	174.8	0.9452	0.5643	1.674

* ICP-OES (Inductively Coupled Plasma—Optical Emission Spectrometers).

**Table 2:** The cell viability (MTT assay; 3-[4,5-dimethylthiazol-2-yl]-2,5-diphenyl tetrazolium bromide) and the evaluation of the morphology of cells treated with the control (contrast reverse-phase microscope CKX41 (Olympus, Tokyo, Japan), mag. 10×).

Control	Suspension	Cell Viability (%)	Cell Morphology in Culture	Grade
HDPE ** negative	100%	95.59	Discrete intracytoplasmic granules. Lysis of the cells was not observed. The cells cover the entire pit. Many cells in divisions (Figure 4a,b)	0
SLS *** positive	0.10 mg/mL	75.40	More than 20% of cells rounded, shrunk, separating from the substrate without densities of cytoplasm, single cells disrupted. Empty spaces between cells (Figure 5b)	2
	0.15 mg/mL	12.60	Completely destroyed cell culture. Extensive cell lysis (Figure 5c)	4
	0.20 mg/mL	12.50	Completely destroyed cell culture. Extensive cell lysis (Figure 5d)	4

** HDPE – High Density Polyethylene, *** SLS – Sodium lauryl sulfate.

**Table 3:** The cell viability and the evaluation of the morphology of cells treated with the suspensions of the nHAP.

Sample	Suspension (%)	Cell Viability (%)	Cell Morphology in Culture	Grade
nHAP-800	100	87.64	About 10% of the cells shrunk, separating from the substrate, visible intracytoplasmic granules (Figure 6a)	1
	50	87.35	Approximately 15% of cells shrunk, separating from the substrate, visible intracytoplasmic granules (Figure 6b)	1
	25	91.79	Discrete intracytoplasmic granules. Single cells shrunk. Lysis of the cells was not observed (Figure 6c).	0
	12.5	93.37	Discrete intracytoplasmic granules. Lysis of the cells was not observed. Culture density was comparable to the density of control culture, and there were many cells in divisions (Figure 6d).	0
nHAP-900	100	88.87	Approximately 15% of cells shrunk, separating from the substrate, visible intracytoplasmic granules (Figure 7a)	1
	50	95.59	Discrete intracytoplasmic granules. Lysis of the cells was not observed. Culture density was comparable to the density of negative control culture (Figure 7b).	0
	25	95.00	Discrete intracytoplasmic granules. Lysis of the cells was not observed. Culture density was comparable to the density of negative control culture (Figure 7c).	0
	12.5	92.48	Discrete intracytoplasmic granules. Lysis of the cells was not observed. Culture density was comparable to the density of control culture (Figure 7d).	0
nHAP-1000	100	88.30	Approximately 10% of cells shrunk, separating from the substrate, visible intracytoplasmic granules (Figure 8a)	1
	50	94.76	Discrete intracytoplasmic granules. Lysis of the cells was not observed. Culture density was comparable to the density of negative control culture (Figure 8b).	0
	25	100.10	Discrete intracytoplasmic granules. Lysis of the cells was not observed. Culture density was comparable to the density of negative control culture (Figure 8c).	0
	12.5	104.40	Discrete intracytoplasmic granules. Lysis of the cells was not observed. Culture density was comparable to the density of control culture (Figure 8d).	0

**Table 4:** Criteria of toxicity effect based on changes in cell morphology [12].

Grade	Toxicity	Cell Morphology
0	lack	Discrete intracytoplasmic granules, no evidence of cell lysis, lack of inhibition of cell growth
1	weak	No more than 20% of rounded, shrunk cells, separating from the substrate without densities of cytoplasm, individual cells disrupted
2	moderate	No more than 50% of rounded cells, no evidence of granules, vast cell lysis and empty spaces between cells
3	average	No more than 70% of rounded cells, cells underwent lysis
4	strong	Almost completely or completely damaged cell culture
